# Real-time assessment of exercise rehabilitation and symptoms in hospitalized COPD patients

**DOI:** 10.3389/fmed.2026.1858176

**Published:** 2026-06-18

**Authors:** Rong Cheng, Hui Wu, Peiran Guo, Chuqin Xiong, Zhixia Zhang

**Affiliations:** 1 Nursing Department, Tianyou Hospital Affiliated to Wuhan University of Science and Technology, Wuhan, Hubei, China; 2 Hubei Province Key Laboratory of Occupational Hazard Identification and Control, Institute of Nursing Research, Wuhan University of Science and Technology School of Medicine, Wuhan, Hubei, China

**Keywords:** chronic obstructive pulmonary disease, ecological momentary assessment, exercise, linear model, symptom burden

## Abstract

**Objective:**

Using ecological momentary assessment (EMA), this study aims to examine real-time associations between exercise rehabilitation and symptoms in hospitalized middle-aged patients with COPD and to explore the moderating role of self-efficacy.

**Methods:**

In this study, 168 middle-aged patients with COPD (mean age = 50.91 ± 4.27 years) underwent 7-day monitoring using EMA. Patients assessed dyspnea, irritability, and active exercise rehabilitation behavior three times daily via mobile devices. A multilevel linear model was used to analyze the dynamic relationship between active exercise rehabilitation behavior and symptoms, as well as the moderating effect of self-efficacy.

**Results:**

Dyspnea (*β* = −0.928, *p* < 0.001) and irritability (*β* = −0.469, *p* = 0.004) significantly predicted low active exercise rehabilitation behavior, while self-efficacy positively predicted active exercise rehabilitation behavior (*β* = 0.319, *p* = 0.001). In addition, self-efficacy significantly buffered the negative effect of irritability on active exercise rehabilitation behavior (*β* = −0.039, *p* < 0.05).

**Conclusion:**

Active exercise rehabilitation behavior is closely related to symptoms, and self-efficacy shows promising value in improving active exercise rehabilitation behavior. Clinical staff must dynamically manage patient clinical symptoms and incorporate self-efficacy into pulmonary rehabilitation programs to protect patients’ active exercise rehabilitation behavior from the impact of negative emotional fluctuations.

## 1 Introduction

Chronic obstructive pulmonary disease (COPD) is the third leading cause of mortality worldwide (1, 2). The Global Burden of Disease (GBD) 2021 study (3) showed that there are 3.5 million COPD-related deaths globally, with a projected surge to 5.4 million by 2060. In China, the prevalence of COPD in the middle-aged population is as high as 13.7%, which is higher than the global average of 12.38% in the same age group (4). Most patients face a rapid decline in daily activities and lung function (5). Notably, the 2021 Global Initiative for Chronic Obstructive Lung Disease (GOLD) guidelines recommend pulmonary rehabilitation as the primary non-pharmacological treatment for COPD, with the aim to enhance patients’ physical/mental well-being and foster sustained health-promoting behaviors (6).

Pulmonary rehabilitation (PR) is an important non-pharmacological therapy, with exercise training forming its cornerstone (7). Active exercise has been shown to enhance exercise capacity, quality of life, and reduce dyspnea compared with passive exercise (8, 9). Active exercise rehabilitation behavior refers to voluntary, structured, and repetitive physical activities performed by patients to maintain or improve their physical function, including walking, standing up and squatting, and bodyweight resistance training (10). Unlike passive rehabilitation methods (such as therapist-guided activities), active exercise rehabilitation emphasizes initiative, goal-orientation, and patient-driven effort. Furthermore, in-hospital rehabilitation is a core aspect of the transitional management of patients with COPD, reducing hospital stays by an average of 4.27 days and significantly improving post-discharge exercise adherence and long-term quality of life (11). However, only 26 to 30% of patients with COPD fulfill the World Health Organization (WHO) physical activity recommendations (12), with a high rate of exercise interruption of 60.3%, especially during the hospital-home transition phase (13).

Previous studies have shown that daily symptom burden has an important impact on health status and difficulties with exercise training (1). Dyspnea is a characteristic symptom of COPD, and up to 82% of patients with COPD have severe dyspnea (14). To manage breathlessness, patients with severe dyspnea frequently restrict daily movement, inadvertently reinforcing a detrimental spiral of declining activity, muscle atrophy, and accelerated disease progression (10, 15). Among psychosocial factors, irritability is a highly prevalent symptom among people with COPD (42%) (16). More patients with low exercise levels have severe negative emotions than those with moderate or high exercise levels (17). A multicenter study prospectively demonstrated that higher negative emotions scores predicted fewer steps per day and a decline in overall activity levels over time among COPD patients, independent of baseline physical activity and disease severity (18).

Self-efficacy is conceptualized as a control belief that refers to confidence in one’s ability to carry out behavior needed to reach a desired outcome (19). Social Cognitive Theory proposes that self-efficacy influences active exercise rehabilitation behavior both directly and through mediating factors like goal setting, self-regulation, facilitators, barriers, and outcome expectations (19). A study by Song et al. (20) indicated that self-management interventions focused on improving self-efficacy significantly enhanced exercise capacity and quality of life, while also reducing rehospitalization rates among patients with COPD. However, previous studies have relied on retrospective designs, failing to capture daily fluctuations in symptom burden and rehabilitation behaviors, while neglecting the concurrent assessment of motivational factors such as self-efficacy. A comprehensive dynamic influence of these factors on daily symptoms and rehabilitation adherence could enable real-time intervention adjustments during high-risk transition periods, ultimately improving long-term adherence and functional outcomes.

Ecological momentary assessment (EMA) can leverage mobile technology to capture real-time data on behavior, cognition, and affect within naturalistic settings (21). Compared with traditional intermittent assessments, the EMA approach dynamically captures patient activity patterns and symptom burden during hospitalization, revealing individual differences and dynamic interrelationships between these factors. In recent years, its application in COPD research has grown considerably. Davies et al. (22) co-developed with patients an EMA app to assess symptoms including dyspnea, coughing, tiredness, and sleep quality. This study not only confirmed the acceptability of EMA among COPD patients but also demonstrated the potential of this approach in symptom-triggered interventions. In addition, while our previous EMA studies delineated dynamic associations between key symptoms and physical activity in COPD patients (10), the underlying psychological mechanisms remain largely unexplored.

Building on this foundation, the present study proposes an integrative “physiological – psychological” framework, incorporating core psychological variables and positioning self-efficacy as a pivotal moderator. We not only examined the combined predictive effects of dyspnea (a physiological symptom) and irritability (a psychological symptom) on active exercise rehabilitation behavior but also specifically elucidated how self-efficacy modulates the pathway from symptom perception to behavioral decision-making. Our results provide empirical evidence for the clinical construction of a transitional stratified management system and precise rehabilitation strategies in the treatment of COPD.

## 2 Materials and methods

### 2.1 Participants

Patients were recruited from the Department of Respiratory and Critical Care Medicine at a tertiary general hospital in Wuhan, Hubei Province, China, identified through electronic medical records from September 30, 2024, to January 15, 2025. We applied following inclusion criteria: (1) COPD diagnosis according to GOLD criteria (23); (2) age 45–59 years, meeting the WHO’s definition of middle age (24); (3) Barthel Index score of > 41 [this was to ensure that all participants retained sufficient functional independence to participate in exercise-based rehabilitation (25)]; (4) able to safely participate in physical activity; and (5) the patient owns a smartphone, has access to the Internet, and can complete the EMA recording after training. The exclusion criteria were as follows: (1) restrictive lung disease or asthma; (2) major cardiac, pulmonary, hepatic, renal, or psychiatric disorders; (3) comorbidity with cognitive and communication disorders; and (4) comorbidity with severe skeletal musculoskeletal pathology or other disorders that affect exercise function. Prior to testing, all participants provided informed consent. Detachment criteria included the following: (1) transfer to another hospital, department, or death due to other diseases during the study; (2) withdrawal from the study for various reasons or poor compliance during the study; and (3) EMA data completeness <80% or data quality failure. All study procedures involving human participants complied with the Declaration of Helsinki.

This study measured each participant 21 times (7 days × 3 measurements). Sample size was determined using a single-group repeated measures design. Using G-Power software, it was calculated that under the assumptions of a confidence level of 1-*β* = 0.95, an average correlation coefficient of *r* = 0.5, an effect size of *f* = 0.14 (small effect), and a significance level of *α* = 0.05, while accounting for a 20% non-response rate, the minimum required sample size was 56 participants. Additionally, to ensure the robustness and reliability of the results from the new multilevel model (26), this study increased the required sample size to 130 cases.

### 2.2 Procedures

This longitudinal prospective study used EMA methodology. Within 24 h of admission due to acute exacerbation of COPD (AECOPD), study participants completed the baseline survey with researcher assistance, which included a demographic questionnaire and the Chinese version of the Pulmonary Rehabilitation Adapted Index of Self-Efficacy (PRAISE) scale. Prior to enrollment in the 7-day EMA monitoring phase, all participants were in stable clinical condition and had received routine PR health education from their attending physicians and nurses.

The literature suggests that 7-day period is typically reflective of habitual activity patterns (11). Therefore, this study conducted a 7-day EMA. This study employed a hybrid EMA sampling strategy that integrates event- and time-based protocols. All EMA prompts were sent as SMS text messages containing a personalized survey link. Each EMA recording required approximately 3–5 min to complete. For event-based sampling, participants could initiate daily active exercise rehabilitation behavior assessment at any time following the occurrence of the target behavior via a text message-delivered survey link, with no predefined time constraints on completion. For time-based sampling, participants received text message prompts at fixed daily intervals (7:00 a.m., 12:00 a.m., and 20:00 p.m.), each initiating a 2-h response window. This daily prompting frequency of three times a day is consistent with prior research (27), and was implemented to minimize participant dropout and burden. To monitor compliance, the researchers checked the EMA’s response status three times a day (at 9:00 a.m., 2:00 p.m., and 10:00 p.m.) via the backend. If no submission was recorded within the initial 2-h window following a prompt, automated reminder text messages were delivered at 30-min intervals, or the responsible nurse reminded patients to fill out the EMA questionnaire in a timely manner during the shift handover and ward rounds. For technical issues (such as links not opening, difficulty navigating the system, or a dead phone battery), participants can immediately contact the research team via the dedicated hotline. Researchers will provide remote assistance within 30 min to resolve the issue, or a clinical nurse will assist the participant in completing the questionnaire using a mobile device provided by the hospital. Records not submitted within the designated 2-h window were classified as missing data for that specific assessment occasion.

### 2.3 Measures

#### 2.3.1 Baseline

At baseline, each participant completed a paper-based questionnaire developed by the researchers to assess sociodemographic and disease-related variables, including sex, age, education, marital status, disease duration, complications, and family history. We then used the Chinese version of the Pulmonary Rehabilitation Adapted Index of Self-Efficacy (PRAISE) scale, a well-established instrument for assessing self-efficacy in PR across diverse cultural and clinical settings. Originally developed by Vincent et al. (28) in 2011 and subsequently cross-culturally adapted and validated in Chinese by Ye et al. (29) in 2023, the scale has been widely employed in local studies to measure self-efficacy among chronic disease patients undergoing rehabilitation (30). The PRAISE scale consists of 13 items, including five items in the self-efficacy dimension, five in the coping ability dimension, and three in the exercise self-efficacy dimension. Each item was rated on a four-point Likert scale, ranging from 1 = “completely disagree” to 4 = “completely agree,” and the scale was rated from 13 to 52, with higher scores indicating higher self-efficacy. Cronbach’s alpha for the original scale was 0.896, and Cronbach’s alpha in this study was 0.922.

#### 2.3.2 EMA

Assessment for active exercise rehabilitation: This assessment questionnaire was compiled by the researcher and included the type of active exercise, number of exercise sessions, total duration of active exercise (in minutes). For the “Type of active exercise” section, patients may select from the following options: walking, seated stepping, ankle flexion and extension, upper-body resistance training with resistance bands, standing and squatting, other, and provide a description. This assessment was self-reported by the patients based on the actual completion of exercise rehabilitation.

Assessment for symptom: Using a Visual Analog Scale (VAS), we then examined current feelings of dyspnea (“How hard are you breathing at the moment [0 = not at all breathless, 10 = extremely breathless]?”). We then used another VAS to examine current feelings of irritability (“How irritable do you feel at the moment [0 = not irritable at all, 10 = very irritable]?”). The VAS is widely used to assess dyspnea in COPD (21) and irritability (31).

### 2.4 Quality control

To ensure that participants correctly understood and assessed the types of active exercise and the severity of symptoms, the researchers provided participants with a detailed description of the study and the meaning of the EMA questionnaire. Upon enrollment, all participants received a guide containing both illustrations and text that detailed the specific components of each exercise. Participants were simultaneously instructed on completion of the EMA surveys. Additionally, data from participants who withdrew midway or failed to complete 80% or more of the EMA prompts during the 7-day EMA period were recorded as invalid data. In the data analysis stage, we assumed that missing data were missing at random (MAR) and used full-information maximum likelihood estimation (FIML) in Mplus to handle missing data, without performing any manual imputation.

### 2.5 Statistical analysis

Firstly, descriptive analyses (including mean and frequency) were conducted on participant characteristic data using SPSS 23.0 (IBM Corp., Armonk, NY, USA). Subsequently, univariate analyses were conducted on demographic characteristics with the duration of active exercise rehabilitation behavior as the dependent variable. Significant variables were incorporated as covariates in subsequent multilevel linear models. Finally, we employed Mplus 8.3 software (Muthén & Muthén, Los Angeles, CA, USA) to construct multilevel linear models using a hierarchical approach with block entry to analyze nested data, and employed FIML estimation to handle missing data. We first calculated an empty model (with no predictors) to estimate an intraclass correlation coefficient 1 (of 1). Subsequently, a Level 1 model was established to examine the predictive effects of dyspnea and irritability on active exercise rehabilitation behavior. All independent variables in the Level 1 model were centered on group means to distinguish between within-subject and between-subject effects. To capture trends and correlations in repeated-measure data over time, we included time as a covariate in the Level 1 model to account for linear or nonlinear trends in active exercise rehabilitation behaviors during hospitalization. Thereafter, a Level 2 model was established to examine the predictive effects of PRAISE on active exercise rehabilitation behavior. All independent variables in the second-order model were centered on the mean to test the between-subject effect of PRAISE on active exercise rehabilitation behavior. Finally, we developed a mixed-effects model that included both Level 1 and Level 2 variables to examine the combined predictive effects of dyspnea, irritability, and PRAISE on active exercise rehabilitation behavior and the moderating effects of PRAISE. All models were estimated using the maximum likelihood (ML) method, and the level of statistical significance was set at *p* < 0.05.

## 3 Results

### 3.1 Descriptive statistics

In total, 178 participants consented to participate in the study, with a final sample of 168 participants (94.4%). Ten participants dropped out due to a change in their condition during the study. These 10 dropouts were excluded from all analyses because follow-up EMA data were missing after their condition changed. Thus, the final analysis sample consisted of 168 participants who completed the full 7-day EMA protocol. This exclusion did not affect the temporal continuity of the remaining participants’ longitudinal data, as all 168 participants had a full schedule of 21 EMA prompts (7 days × 3 times daily), and no participant dropped out after enrollment. Although there were occasional missing responses during the EMA monitoring, no participant had a complete block of consecutive missing days. Of the 168 participants, 94 (56.0%) were male, 74 (44.0%) were female; 160 (95.2%) were married, 6 (3.6%) were unmarried and 2 (1.2%) were divorced; the mean age was 50.91 ± 4.27 years. Table 1 presents the sample characteristics. In addition, the total score on the PRAISE scale was 28.69 ± 8.30; the scores of the three dimensions were as follows: general self-efficacy, 11.13 ± 3.31; coping ability, 10.87 ± 3.66; and exercise self-efficacy, 6.69 ± 2.17.

**Table 1:** Demographic and primary variables of the study participants (*n* = 168).

Variables	*n*	%	*t*/r/F	*p* values
Sex			−3.431	0.002
Male	94	56.0		
Female	74	44.0		
Age (years), Mean (SE)	50.91 ± 4.27		−0.214	0.121
Ethnic group			1.962	0.128
Majority ethnic group	164	97.62		
Minority ethnic group	4	2.38		
Education			3.400	0.016
Illiteracy	4	2.3		
Primary school	9	5.4		
Junior high school	123	73.2		
High school	27	16.1		
Undergraduate or above	5	3.0		
Marital status			1.838	0.181
Unmarried	6	3.6		
Married	160	95.2		
Divorced	2	1.2		
Home location			−1.767	0.083
Rural	129	76.7		
Urban	39	23.3		
Comorbidities			0.199	0.843
Yes	29	17.3		
No	139	82.7		
Caregiver			0.466	0.707
Living alone	37	22.0		
Children	5	3.0		
Partner	120	71.4		
Carer or other family member	6	3.6		
Disease duration (years)			1.956	0.133
≤10	90	53.6		
11–20	47	28.0		
21–30	15	8.9		
≥31	16	9.5		
Family history			−0.389	0.699
Yes	20	11.9		
No	148	88.1		
Smoking			3.077	0.055
Current smoking	20	11.9		
Never	47	28.0		
Quit smoking	101	60.1		
GOLD			1.956	0.133
I	14	8.3		
II	104	62.0		
III	37	22.0		
IV	13	7.7		
Episodes of AECOPD (within the past year)			−0.196	0.846
Yes	122	72.6		
No	46	27.4		
FEV1 (L), Mean (SE)	1.55 ± 0.57		−0.054	0.696

SE, standard error; GOLD, Global Initiative for Chronic Obstructive Lung Disease; AECOPD, acute exacerbation of chronic obstructive pulmonary disease; FEV1, forced expiratory volume in 1 s.

### 3.2 EMA completion rate

First, to test the hypothesis regarding missing values, we compared the missing rates across different number of days and different time points. The results showed no significant differences in missing rates across different days periods (range = 12.30–14.09%) or time points (range = 12.93–13.10%) (*p* > 0.05), supporting the MAR assumption.

Over the course of the 7-day EMA study period, participants were provided with a mean of 3,528 symptom assessment surveys, of which 3,069 (87.0%) had effective responses (missing rate 13.0%). Participants completed a mean of 2.61 EMA surveys per day (standard deviation [SD] = 0.22, range = 2.17–2.95), indicating excellent compliance. In addition, during the 7-day EMA monitoring period, the total number of reports on event-based active exercise reports was 197, 275, 269, 342, 306, 348, and 313, respectively.

### 3.3 Trends in active exercise rehabilitation behavior and symptom burden

**Figure 1:**
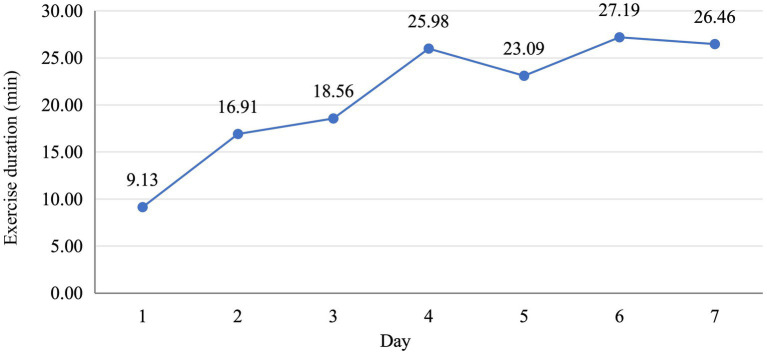
Participant self-reported mean daily hours of active exercise rehabilitation behavior.

During the self-reporting periods, participants completed 21.04 (SD = 6.59) minutes of active exercise rehabilitation per day (Figure 1). In addition, the mean daily scores for dyspnea and irritability symptoms were 3.67 (SD = 1.74) and 3.50 (SD = 1.75), respectively (Table 2).

**Table 2:** Momentary intensity of patient self-reported symptom burden during 7-day ecological momentary assessment [(score, x̄ ± SD), *n* = 168].

Symptom burden	Day 1	Day 2	Day 3	Day 4	Day 5	Day 6	Day 7
Dyspnea	4.57 ± 1.70	4.02 ± 1.74	4.17 ± 1.63	3.57 ± 1.82	3.48 ± 1.55	3.07 ± 1.58	2.98 ± 1.51
Irritability	3.81 ± 1.80	3.78 ± 1.93	3.12 ± 1.45	4.14 ± 1.86	3.15 ± 1.68	3.24 ± 1.67	3.36 ± 1.58

SD, standard deviation.

### 3.4 One-way analysis of variance

Before constructing the multilevel model, we conducted univariate analyses using the duration of active exercise rehabilitation behavior as the dependent variable and incorporated significant variables as covariates in subsequent models. Results indicated that sex (*t* = −3.431, *p* = 0.002) and educational attainment (*F* = 3.400, *p* = 0.016) showed significant positive associated with the duration of active exercise rehabilitation behavior. The results of the univariable analyses are shown in Table 1.

### 3.5 Multilevel models

#### 3.5.1 Model 1: zero model

Before establishing multilevel models, the intraclass correlation coefficient 1 (ICC 1) was computed. The result thereof was 0.293 (>0.059), which meant that the data met the requirement of multilevel regression analysis and that the sources of individual differences should be analyzed in a stratified manner.

#### 3.5.2 Model 2: associations between intra-level variables and active exercise rehabilitation behavior

Table 3 shows the results of the associations among dyspnea, irritability, and active exercise rehabilitation behavior. Higher levels of dyspnea (*β* = −0.913, *p* < 0.001) and irritability (*β* = −0.360, *p* < 0.05) were significantly associated with shorter exercise duration. In addition, sex was also associated with active exercise rehabilitation behavior (*β* = 3.411, *p* < 0.001), with female patients showing longer exercise duration in our sample.

**Table 3:** Associations between intra-level variables and active exercise rehabilitation behavior.

Variables	*β*	SE	*t*	*p* values	95% CI	
					Lower	Upper
Fixed effects						
Intercept	7.015	0.529	13.250	<0.001	5.977	8.053
Layer 1 variables						
Time	0.215	0.027	7.998	<0.001	0.162	0.267
Sex	3.411	0.947	3.601	<0.001	1.554	5.267
Education	0.203	0.480	0.423	0.673	−0.738	1.144
Dyspnea	−0.913	0.182	−5.008	<0.001	−1.270	−0.556
Irritability	−0.360	0.152	−2.366	0.018	−0.658	−0.062
Random effects						
Intercept	13.636	1.717	7.941	<0.001	10.271	17.002

Coef., coefficient; SE, standard error; CI, confidence interval.

#### 3.5.3 Model 3: associations between inter-level variables and active exercise rehabilitation behavior

Table 4 shows the association between self-efficacy (the PRAISE) and active exercise rehabilitation behavior. The results indicated that higher levels of self-efficacy (*β* = 0.336, *p* < 0.001) were significantly associated with longer exercise duration, suggesting that participants with greater self-efficacy tend to engage in longer durations of active exercise rehabilitation behavior.

**Table 4:** Associations between inter-level variables and active exercise rehabilitation behavior.

Variables	*β*	SE	*t*	*p* values	95% CI	
					Lower	Upper
Fixed effects						
Intercept	7.015	0.388	18.083	<0.001	6.255	7.775
Layer 2 variables						
Sex	1.399	0.801	1.747	0.081	−0.170	2.969
Education	0.277	0.371	0.748	0.454	−0.449	1.004
PRAISE	0.336	0.040	8.430	<0.001	0.258	0.414
Random effects						
Intercept	6.398	1.516	4.220	<0.001	3.427	9.369

Coef., coefficient; SE, standard error; CI, confidence interval; PRAISE, scores of the Pulmonary Rehabilitation Adapted Index of Self-Efficacy.

#### 3.5.4 Model 4: associations between inter- and intra-level variables and active exercise rehabilitation behavior and the moderating effects of PRAISE

Table 5 shows the results of the prediction of dyspnea, irritability, and PRAISE scale on active exercise rehabilitation behavior and the moderating effect of the PRAISE scale. The results showed that higher levels of dyspnea and irritability were significantly associated with shorter exercise duration (*p* < 0.05). In addition, self-efficacy was significantly positively correlated with exercise duration (*p* = 0.001). More importantly, a significant moderating effect of self-efficacy was also observed on the relationship between irritability and exercise behavior (*p* = 0.047), suggesting that self-efficacy may attenuate the negative influence of irritability in active exercise rehabilitation behavior.

**Table 5:** Associations between inter- and intra-level variables and active exercise rehabilitation behavior and the moderating effects of PRAISE.

Variables	*β*	SE	*t*	*p* values	95% CI	
					Lower	Upper
Fixed effects						
Intercept	7.015	0.388	18.064	<0.001	6.254	7.776
Layer 1 variables						
Time	0.088	0.327	0.268	0.789	−0.553	0.729
Sex	1.588	1.314	1.208	0.227	−0.989	4.164
Education	0.290	0.353	0.822	0.411	−0.401	0.981
Dyspnea	−0.928	0.173	−5.363	<0.001	−2.896	−0.288
Irritability	−0.469	0.162	−2.904	0.004	−0.728	−0.174
Layer 2 variables						
PRAISE	0.319	0.096	3.340	0.001	0.132	0.506
Moderating effect						
PRAISE × Dyspnea	−0.024	0.023	−1.038	0.299	−0.070	0.022
Sex × Dyspnea	−0.879	0.538	−1.634	0.102	−1.933	0.175
Education × Dyspnea	−0.079	0.130	−0.605	0.545	−0.333	0.176
PRAISE × Irritability	−0.039	0.020	−1.989	0.047	−0.078	−0.001
Sex × Irritability	0.238	0.618	0.385	0.700	−0.973	1.449
Education × Irritability	0.067	0.103	0.650	0.516	−0.136	0.270
Random effects						
Intercept	6.546	1.754	3.731	<0.001	3.107	9.984

Coef., coefficient; SE, standard error; CI, confidence interval; PRAISE, scores of the Pulmonary Rehabilitation Adapted Index of Self-Efficacy.

## 4 Discussion

We examined within-subject associations between daily symptom burden and active exercise rehabilitation behavior in patients with COPD during hospitalization through an EMA, expanding on previous studies by including self-efficacy. Within-subject daily changes in symptom burden may be more strongly related to active exercise rehabilitation behavior than between-subject differences. Our findings also suggest that self-efficacy effectively weakens the inhibitory effects of irritability on active exercise rehabilitation behavior. These findings are key to achieving accurate rehabilitation nursing in transition, while promoting the transition of our PR intervention model from static to dynamic adaptive management.

Objective assessment of patient active exercise rehabilitation behavior showed that participants exercised for a mean of 21.04 min over the 7-day period, which is approximately 1.5 times as much as the amount of exercise performed by older patients with COPD (13.9 ± 15.2) as investigated by O′ Neill et al. (32). This disparity may be attributable to the fact that our study consisted of middle-aged patients who likely possessed greater social participation needs and more active rehabilitation intentions. In addition, during the initial 3 days, participants generally exhibited low exercise durations alongside a relatively high symptom burden. Exercise duration peaked on day 4, followed by a fluctuating downward trend, and symptom burden scores began to show gradual improvement. The results of this study distinguish it from previous studies and innovatively use EMA research methodology to objectively and immediately capture the dynamic change curves of active exercise rehabilitation behavior and symptom burden. These results can help healthcare professionals provide timely interventions for AECOPD and stage-specific rehabilitation needs and prevent the decline of exercise levels and exercise effect over time after discharge.

The results of our study suggest that dyspnea is significantly and negatively associated with active exercise rehabilitation behavior (*β* = −0.928, *p* < 0.001). As the most prevalent symptom of COPD, dyspnea reduces exercise engagement by creating an imbalance between the demand for breathing and the ability to achieve that demand (33). Esteban et al. (34) demonstrated that higher dyspnea scores in hospitalized patients with COPD were associated with an average reduction in exercise time of between 14.15–27.4 min that day. Furthermore, Mille et al. (35) established that a 6-week smartphone-based EMA symptom monitoring program enhanced self-management behaviors and health competence in patients with COPD. These studies underscore the significant relationship between dyspnea and decreased daily active exercise rehabilitation behavior during PR management. Therefore, future research could provide EMA-based adaptive interventions for patients via mobile devices. This could help patients identify impending exacerbations during the home-based rehabilitation phase and adjust their exercise program to maintain the long-term effects of exercise training after discharge.

Irritability exhibited a significant negative association with active exercise rehabilitation behavior (*β* = −0.469, *p* = 0.004), consistent with findings from Lee et al. (36). Using EMA, Lee et al. (36) explored the dynamic interplay between mood symptoms and exercise patterns and found that patients experienced significant decreases in physical activity levels after experiencing adverse emotions, such as irritability and anxiety. Psychological symptoms in patients with COPD can heighten dyspnea perception, contributing to increased exercise-related breathlessness, diminished exercise tolerance, and reduced quality of life (18). Melhem et al. (37) further revealed that emotional burden exceeds physiological burden in COPD, with high-severity psychological symptoms affecting 66 to 88% of patients. Given the highly diverse symptom experiences among patients with COPD, mobile device-based EMA could be relied upon in the future to assess and manage physical and psychological symptoms, rather than relying on the assessment of respiratory symptoms alone. This will help to better identify individual rehabilitation needs, optimize treatment and care, and ensure the long-term continuation of PR outcomes.

In addition, our study indicated a significant positive association between self-efficacy and active exercise rehabilitation behavior (*β* = 0.319, *p* = 0.001). According to self-efficacy theory (19), individuals with stronger self-efficacy are more likely to perceive benefits during exercise, thereby enhancing adherence to health-promoting behaviors through psychological regulation. Supporting this finding, Selzler et al. (38) demonstrated that self-efficacy in COPD is correlated with functional exercise capacity and physical activity, although the measurement instruments influence the strength of this relationship. Additionally, we observed that the negative association between irritability and active exercise rehabilitation behavior was weaker among patients with higher self-efficacy (*β* = −0.039, *p* < 0.05). Bandura (19) suggested that self-efficacy can modulate final behavioral decisions by manipulating the expected success of emotion regulation and that self-efficacy can be improved through training. However, it should be noted that while this study identified a moderating effect of self-efficacy on the relationship between symptoms and behavior, factors such as disease severity, treatment status, rehabilitation resources, and social support may all influence the strength of this association (13). Therefore, healthcare professionals should establish a multidimensional assessment system to accurately evaluate the potential factors affecting physical activity in different patient groups and encourage patients to gradually transition from passive to active rehabilitation in order to promote sustained improvements in pulmonary rehabilitation outcomes.

This study has several limitations. First, although the study was powered to examine within-person effects, our sample size was relatively small, recruited from a single center, potentially decreasing generalizability to other populations. Larger-scale multicenter research with expanded sample size and increased participant diversity can be conducted in future to validate these findings. In addition, while our statistical models controlled for known confounders (e.g., sex, education level), residual confounding cannot be entirely ruled out. Future large-scale, multicenter prospective cohort studies are recommended to proactively collect and adjust for a broader range of potential confounding variables, thereby enabling the development of personalized, adaptive real-time intervention protocols.

## 5 Conclusion

Overall, our findings demonstrated a gradual yet fluctuating trajectory of active exercise rehabilitation activity participation during hospitalization. Significant intra-individual variability exists between symptom burden and active exercise rehabilitation behavior, with self-efficacy playing a crucial buffering role—higher self-efficacy mitigates the negative impact of irritability on active exercise rehabilitation behavior. These findings directly inform strategies to optimize the transition from hospital to home. Developing a phased adaptive plan based on fluctuations in a patient’s exercise behavior during hospitalization can minimize rehabilitation disruption after discharge while maintaining and consolidating the rehabilitation gains achieved during the inpatient period, thereby promoting long-term functional improvement. Simultaneously, integrating self-efficacy enhancement strategies—such as goal setting, verbal reinforcement, and accumulation of successful experiences—into PR programs can effectively help patients develop the ability to independently manage rehabilitation challenges after discharge, thereby promoting the synergistic improvement of functional independence and mental health levels. Furthermore, mobile-based synchronous symptom- and behavior-monitoring technology provides a scalable framework for home-based telemonitoring and real-time personalized intervention, facilitating a smoother transition to home settings.

## Data Availability

The raw data supporting the conclusions of this article will be made available by the authors, without undue reservation.
